# Fertility outcomes in cows with subclinical endometritis after clinical cure of clinical endometritis

**DOI:** 10.1186/s13620-024-00281-0

**Published:** 2024-10-10

**Authors:** Wojciech Barański, Sławomir Zduńczyk, Dawid Tobolski, Milena Krupa

**Affiliations:** 1 Department of Animal Reproduction with Clinic, Faculty of Veterinary Medicine, University of Warmia and Mazury, ul. Oczapowskiego 14, Olsztyn, 10-719 Poland; 2 Private Veterinary Clinic, Warsaw, Poland

**Keywords:** Cows, Clinical endometritis, Subclinical endometritis, Fertility

## Abstract

Clinical endometritis (CE) is common in post-partum dairy cows and is associated with impaired reproductive performance. The aim of the study was to evaluate the effect of subclinical endometritis (SE) in cows clinically cured of CE on their fertility. The study was performed on 215 Holstein Friesian cows with CE diagnosed by vaginoscopy and ultrasound between 21 and 28 days after parturition. All cows were clinically examined three times at an interval of 2 weeks. Cows without signs of CE were considered cured, and endometrial samples from the uteri were collected by cytobrush to diagnose SE using cytological evaluation of polymorphonuclear neutrophils (PMNs) percentage. The threshold for SE was set at ≥ 5% PMNs. Intervals calving to oestrus and calving to conception, first AI pregnancy rate, pregnancy rate 200 days after artificial insemination (AI), the number of AI per pregnancy (AI/P), pregnancy loss, and culling rate were calculated. SE was diagnosed in 40.9% of cows clinically cured of CE. There were significant differences in the AI/P (3.2 vs. 2.6; *p* < 0.027) and the pregnancy loss (18.2% vs. 4.7%; *p* < 0.002) between cows with SE and without SE. Cows with SE showed a tendency towards longer interval calving to conception, lower pregnancy rate 200 days after AI, and higher culling rate. In conclusion, SE after a clinical cure of CE may reduce fertility in dairy cows.

## Introduction

Postpartum uterine inflammatory diseases are common in dairy cows. Uterine infections result from the imbalance between postpartum bacterial contamination of the uterus and uterine defence mechanisms [27, 48]. The inflammation of the uterus is divided into two categories: metritis and endometritis.

In metritis, all layers of the uterine wall show evidence of inflammation. Puerperal metritis is defined as an animal with an abnormally enlarged uterus and a fetid watery red-brown uterine discharge, associated with signs of systemic illness and fever > 39.5 8 C, within 21 days postpartum [47]. The term clinical metritis is used for cows that have delayed involution and a fetid discharge, in the absence of detected fever [49]. Metritis causes significant economic losses due to a decrease in milk production and reproductive efficiency, the cost of treatment, and increased risk of culling [14, 16, 26].

Endometritis is a superficial inflammation of the endometrium. Clinical endometritis (CE) is characterised by the presence of purulent or mucopurulent discharge in the vagina 21 days or more after parturition [28, 46]. The term purulent vaginal discharge (PVD) is also used for clinical endometritis [13, 33]. CE typically occurs in about 20% of cows [13, 28], but in some herds, the incidence of CE is higher [21]. CE is associated with subfertility or infertility and causes large economic losses through the extension of interval calving to conception, increased culling rate, costs of treatment, and reduction in milk production [14, 25, 26].

Subclinical endometritis (SE) is defined by the presence of > 5% of polymorphonuclear neutrophils (PMNs) after 21 days after parturition in endometrial samples obtained using cytobrush in the absence of the clinical signs [31]. However, the threshold of PMNs for diagnosing SE is still under discussion [51]. Subclinical endometritis is also referred to as cytological endometritis [13, 33, 37]. The prevalence of SE varied from 15 to 70% depending on the time of examination in the postpartum period, the threshold for PMNs, and herd-specific factors [2, 19, 24, 31]. Several studies have described impaired reproductive performance in cows affected by SE [3, 8, 19, 24, 31, 50]. However, some studies did not confirm these findings [20, 40, 42, 45].

The relationship between CE and SE is not fully known. It is suggested that CE and SE represent different manifestations of uterine disease [13, 39]. However, cows with CE had a high risk for SE [5, 17, 43, 50].

The impact of SE after the clinical cure of CE on cow fertility has not yet been studied. Thus the aim of the study was to evaluate the effect of subclinical endometritis (SE) in cows clinically cured of CE on their fertility performance.

## Materials and methods

The study was carried out on 800 Polish Holstein Frisian cows from two dairy herds under the herd health program [4] in North-East Poland. The study was approved by the Ethics Committee for Animal Experiments (Approval No. 49/2016). The average milk yield was 9000 L. Cows were housed in a loose housing barn and fed a total mixed ration based on grass and maize silage and supplemented with dairy concentrates, vitamins, and minerals, with unlimited access to water. The feeding ratio was adjusted to the individual demands depending on milk yield by using concentrates in feeding stations. In total, 350 cows in herd A and 450 cows in herd B were examined clinically between 21 and 28 days after parturition to diagnose cows with CE. Cows with retained placenta, metritis, pyometra, acute mastitis, clinical ketosis, or severe lameness were not included in this study.

The examination procedure included inspection of the vulva, tail, and perineum, vaginoscopy, and rectal and ultrasound (Honda 1500 scanner with a 5 MHz linear transducer) examinations of the genital tract. Cows were diagnosed with CE if mucopurulent (< 50% pus) or purulent (> 50% pus) discharge was present in the vagina and uterine horns lumen diameter was larger than 2 mm. CE was diagnosed in 222 cows, which were randomly assigned to one of three groups depending on the treatment method: cephapirin (*n* = 72), PGF2_α_ (*n* = 73), and untreated control (*n* = 77). Cows included in the study were their 2nd to 4th lactation. All cows were clinically examined three times at an interval of 2 weeks. Cows without signs of CE were considered cured. Two cows each from Groups 1 and 3 and three cows from Group 2 were not clinically recovered at the third examination and were excluded from the study so in the final analysis there were 215 cows with CE.

From clinically cured cows endometrial samples were collected by cytobrush (Cervical Rambrush type IC, Shanghai International Holding Corp. GmbH, Germany) to diagnose SE using cytological evaluation of PMNs percentage. The material from the cytobrush was transferred to a microscope slide by rolling the brush on the slide. The smear was treated with cytologic fixative (Cytofix, Samko, Poland), and then all slides were stained using Papanicolau’s method. The percentage of different cell types was calculated by examining 300 visible cells per sample with a light microscope [10, 31]. The smears were evaluated by two different persons blinded to the samples. The threshold for subclinical endometritis was set as equal to or over 5% of PMNs [5, 51].

After the detection of oestrus cows were artificially inseminated (AI) according to “a.m.-p.m.” guidelines. The cows detected to be in oestrus in the morning (a.m.) were submitted for AI that afternoon (p.m.), and cows in oestrus in the afternoon were inseminated the next morning. Pregnancy was diagnosed by ultrasonography 30 days after AI. Cows diagnosed as pregnant were re-examined on day 200 days after AI. The following reproductive performances were calculated for cows with and without SE after clinical cure of CE: intervals calving to oestrus and calving to conception, first AI pregnancy rate, pregnancy rate 200 days after AI, number of AI per pregnancy, pregnancy loss, and culling rate. Pregnancy loss was defined as the percentage of non-pregnant cows 200 days after AI diagnosed 30 days after AI as pregnant. The number of AI per pregnancy (AI/P) was calculated as the total number of AI divided by the number of pregnant cows 200 days after AI.

Statistical analysis was performed using SPSS Statistics 25 software. The normality and homogeneity of the distribution of the parameters were tested using the Shapiro–Wilk and Levene’s tests. The differences in the length of the intervals calving to oestrus and calving to conception and in AI/P were analysed with a one-tailed nonparametric Mann-Whitney U test. The differences in the prevalence of SE and in the first AI conception rate, conception rate 200 days after AI, pregnancy loss, and culling rate were analysed using Fischer’s exact test.

## Results

CE was diagnosed in 88 out of 350 cows (25.4%) in herd A and in 134 cows out of 450 in herd B (29.7%). The difference was not statistically significant (*p* > 0.05). On average, the incidence of CE in both herds was 27.75%. Overall, 215 (96.8%) of the 222 cows with CE were clinically cured within 6 weeks. There were no significant differences in clinical recovery between the treatment groups (*p* > 0.05). The incidence of Se was 35.7% in cows treated with cephapirin, 47.1% in cows treated with PGF2_α,_ and 40.0% in self-cured cows. The difference was not statistically significant (*p* > 0.05). In total, SE was found in 40.9% of clinically cured cows (Table 1).

**Table 1:** Incidence of SE in cows clinically cured of CE according to treatment methods

Variables	Group			Total
	Cephapirin	PGF_2_α	Self-cure	
Clinical cure n/n (%)	70/72 (97.2)	70/73 (95.8)	75/77 (97.4)	215/222 (96.8)
Cured cows with SE n/n (%)	25/70 (35.7)	33/70 (47.1)	30/75 (40.0)	88/215 (40.9)
Cured cows without SE n/n (%)	45/70 (64.3)	37/70 (52.9)	45/75 (60.0)	127/215 (59.1)

**Fig. 1:**
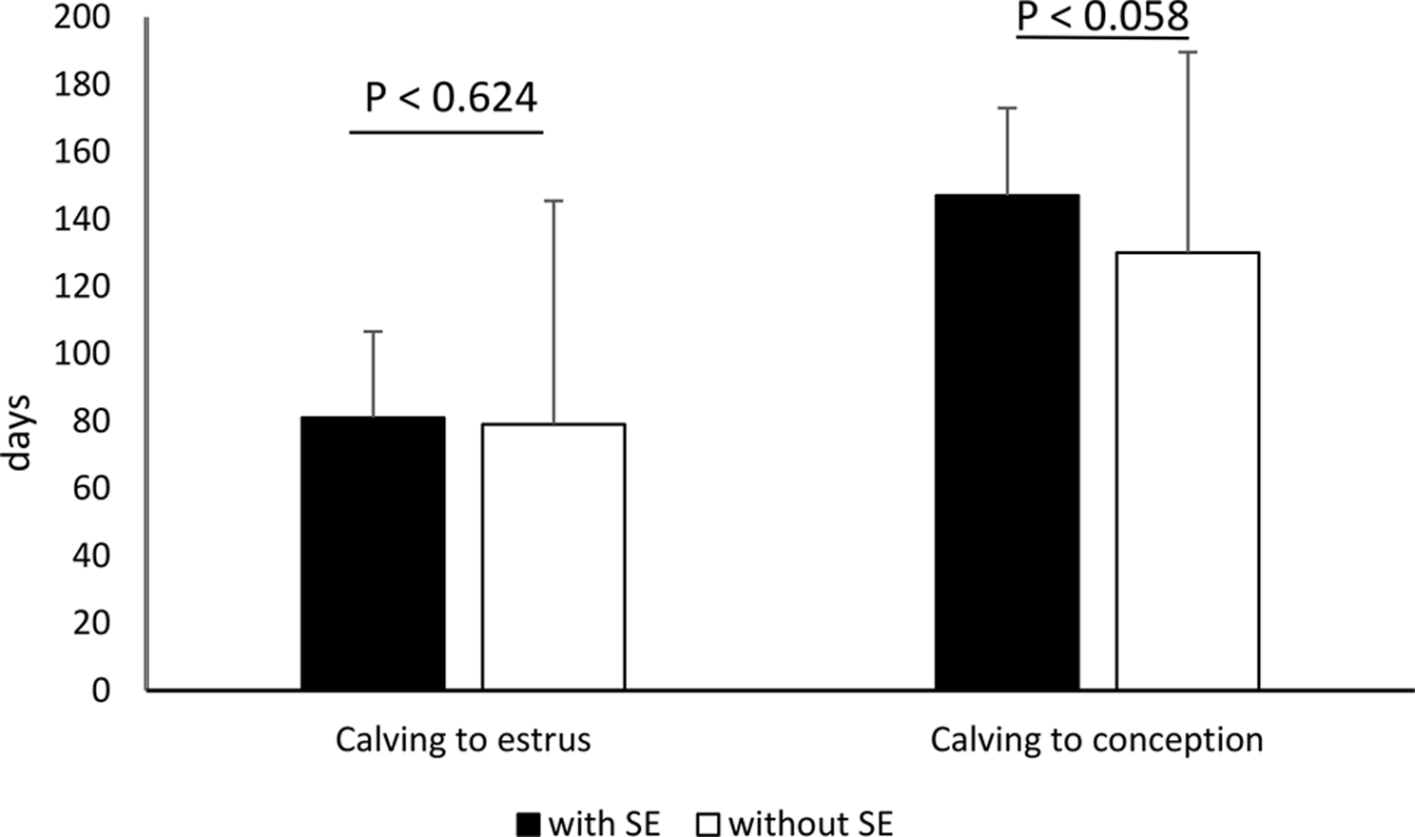
Intervals calving to oestrus and calving to conception (mean ± SD) in cows with and without SE after clinical cure of CE

**Fig. 2:**
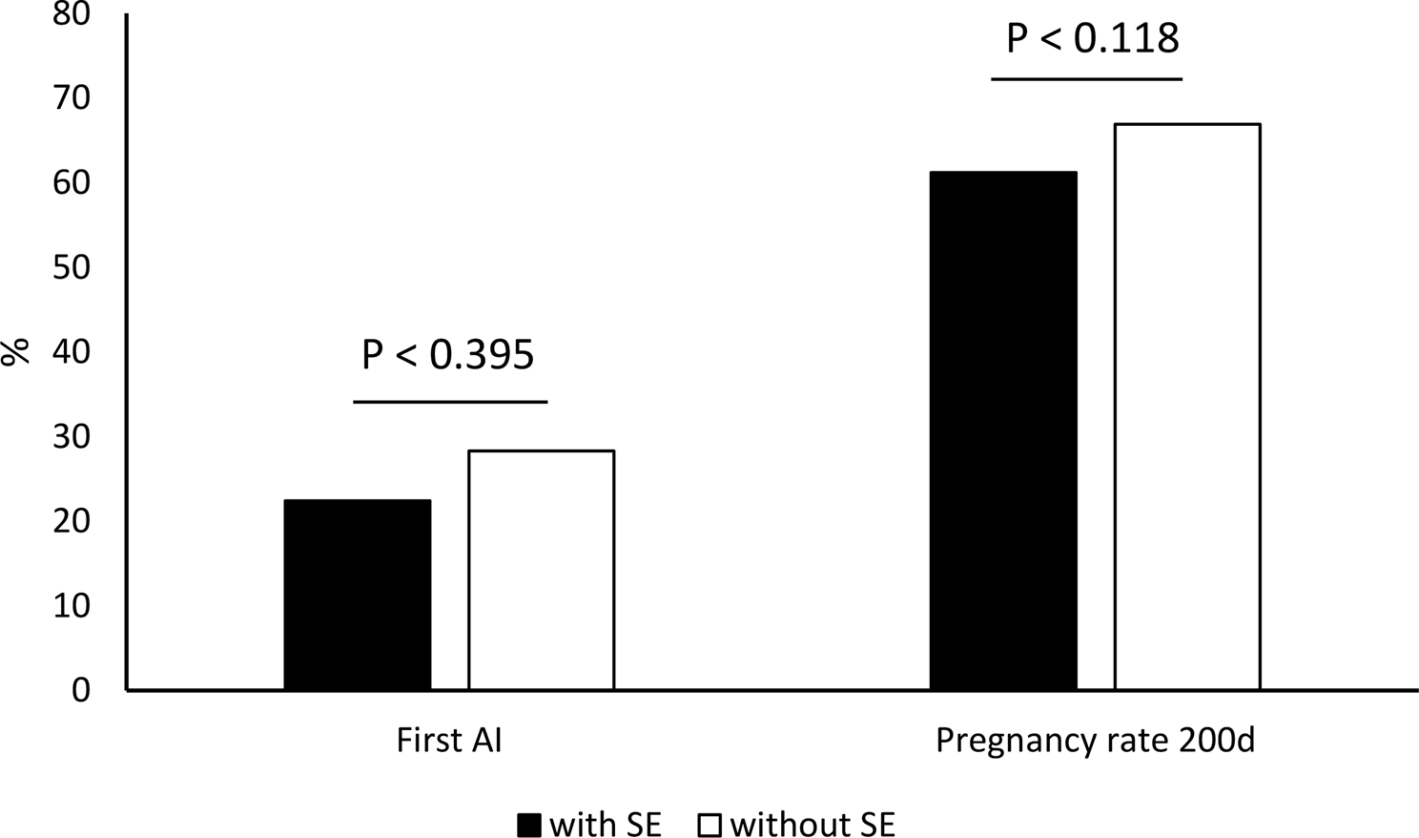
First AI pregnancy rate and pregnancy rate 200 days after AI (%) in cows with and without SE after clinical cure of CE

**Fig. 3:**
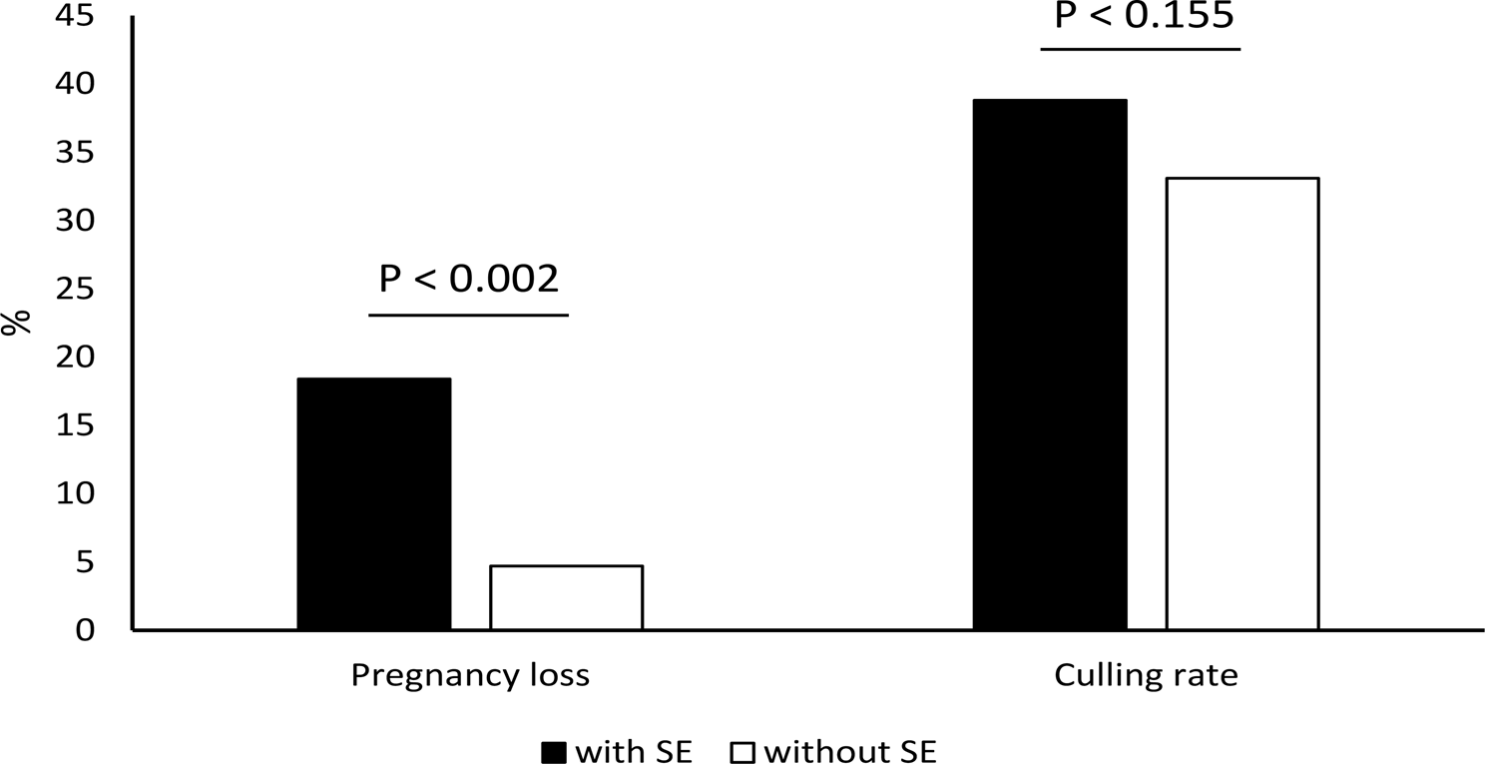
Pregnancy loss (%) and culling rate (%) in cows with and without SE after clinical cure of CE

As there were no differences (*p* > 0.05) in fertility indices between treatment groups, they were analysed jointly for all groups and presented in Figs. 1, 2 and 3. The interval calving to oestrus did not differ statistically between cows with SE and without SE (81.0 ± 25.6 days vs. 79.1 ± 26.0 days; *p* < 0.624). There was no statistical difference in first AI conception rate (22.7% vs. 28.3%; *p* < 0.395), pregnancy rate 200 days after AI (61.4% vs. 66.9%; *p* < 0.118), and culling rate (38.6% vs. 33.1%; *p* < 0.155) between cows with SE and without SE. The AI/P was significantly higher (*p* < 0.027) in cows with SE compared with cows without SE (3.2 vs. 2.6; respectively). The average pregnancy loss was 10.2%. There were significant difference (*p* < 0.002) in pregnancy loss between cows with SE and without SE (18.2% vs. 4.7%). Compared to cows without SE, cows with SE showed a tendency towards longer interval calving to conception (147.0 ± 66.3 days vs. 130.0 ± 59.6 days; *p* < 0.058).

## Discussion

In our study, CE was found in 27.75% of dairy cows. The proportion of cows suffering from CE was within reported ranges [13, 19, 28]. The three treatment groups did not differ concerning the clinical cure of CE. A similar result was reported in another study [15].

The study showed that SE occurs frequently in cows clinically cured of CE. This is in line with studies indicating that cows with CE were more likely to have SE [5, 17, 43, 50]. The pathophysiology of CE and SE in dairy cows is extensively discussed [39, 48, 51]. Uterine inflammation results from the balance between the uterine microbiome (community of microorganisms) and the innate immune response and the regulation of inflammation [27, 48]. The uterus of postpartum cows is usually contaminated with bacteria. These bacteria were eliminated in many cows during the first 5 weeks after parturition, however, in some cows, pathogenic bacteria persist in the uterus as a result of an inadequate immune response and cause inflammation [48]. The most prevalent bacteria cultured from uterine samples of dairy cows with CE were *Trueperella (T.) pyogenes*, *Escherichia (E.) coli*, *Prevotella melaninogenica*, and *Fusobacterium necrophorum* [46]. SE was not associated with the presence of *E. coli* or *T. pyogenes* [42]. Many cows with SE were bacteriologically negative [2, 40]. It seems that in cows with SE uterine infections with known pathogens play a minor role compared with CE [51]. In recent years, culture-independent studies using metagenomic sequencing explored the uterine microbiome of cows with metritis and clinical endometritis. It was shown that the microbiome structure was identical between cows that developed uterine inflammation and healthy cows up until 2 d postpartum. Then there was a shift in the uterine microbiome characterized by a loss of heterogeneity and an increase in *Bacteroidetes and Fusobacteria* in cows with metritis [18] and CE [38]. Uterine bacterial composition was not different between healthy and SE cows, so SE is considered to be a consequence of dysregulation of inflammation rather than changes in the uterine microbiota [27, 38, 39].

The effect of SE after the clinical cure of CE on fertility indices was variable. Interval calving to oestrus in cows after recovery from CE was relatively long with a duration of about 80 days. In healthy dairy cows, the interval calving to oestrus should not exceed 60 days [35]. The prolongation of the interval calving oestrus in this study was the result of CE rather than SE, as it did not differ between cows with SE and without SE. CE significantly increased the risk for delayed ovarian cyclicity before service [16, 32, 36]. The delayed resumption of ovarian activity in cows with uterine inflammation could be associated with the effects of the inflammatory mediators on the hypothalamus and pituitary [48]. Similarly to our results, Gobikrushanth et al. [20] found that the interval from calving to first ovulation was not affected by SE determined at 25 day postpartum. On the contrary, some studies showed that SE > 40 day postpartum in cows without clinical signs of endometritis may delay the establishment of normal estrous cycles [9, 19].

Cows with SE after clinical cure of CE showed a tendency towards lower first AI conception rate, longer interval calving to conception, lower pregnancy rate 200 days after AI, and higher culling rate compared to cows without SE. The conception rates after first AI are usually 40–50% in Polish Holstein-Friesian cows [4]. In this study, first AI conception rate was generally low (< 30%) but not statistically different between cows with SE and without SE. It is consistent with the results of certain studies [17, 20, 34, 40, 42, 45]. On the contrary, in other studies, SE negatively affected first AI conception rate [3, 6, 10, 19, 24, 37].

Several studies reported that SE affects interval calving to conception [3, 6, 8, 17, 19, 24, 31, 50], pregnancy rate [6, 13, 19, 24, 50], and culling rate [19, 24, 50]. In contrast, several studies reported no significant effect of SE on reproductive performance in cows [20, 40, 42, 45]. The effects of CE and SE on reproductive performance were additive. Cows having both CE and SE showed longer interval calving to conception than cows having CE or SE only [13]. The average interval calving to conception in 2003 in Poland for cows under milk recording was 137 days with a recommended voluntary waiting period of 60 days [41]. In our study, the interval calving to conception in cows with SE after clinical cure of CE was 147.0 ± 66.3 days. For Polish Holstein-Friesian, the average number of services per conception of 2.2 was recorded [49]. In our study, the number of AI per pregnancy was higher in cows with SE than in cows without SE after a clinical cure of CE (3.2 vs. 2.6). This was a consequence of the low pregnancy rate after first AI in both groups and high pregnancy loss in cows with SE. About 8–10% of pregnancies in cows are lost between days 30 and 90 of gestation [11, 30]. In our previous study, the pregnancy loss rate between 30 and 260 days in eight dairy herds in northeastern Poland averaged 13.7% [1]. Pregnancy loss is more common among cows with uterine inflammation [29, 43]. In our present study, pregnancy loss was significantly higher in cows with SE after the resolution of CE. This finding is consistent with greater embryonic loss in cows with uterine inflammation as evidenced by a high proportion of PMNs in the uterine lumen [34]. It seems that an inflamed uterine environment impacts embryo quality and survival. SE is associated with local inflammatory reactions resulting in an unfavourable uterine environment for embryo development. In cows with SE, mRNA expression in endometrium and secretion of several proinflammatory cytokines was higher compared with healthy cows [48, 51]. Hill and Gilbert [22] showed that culturing of bovine embryos in media conditioned by exposure to an inflamed endometrium reduced their quality. The high prevalence of SE was reported in repeat breeder cows in some studies [23, 44]. Drillich et al. [12] found that cows with 0% PMN at first AI flushed a significantly higher number of transferable embryos compared to cows with higher endometrial PMNs. However, the embryo survival rate was higher in cows whose proportion of PMN had a slight increase from AI to flushing at day 7. Recently, Barnes et al. [7] showed that recipient beef cows with SE had reduced pregnancy per embryo transfer. However, Ribeiro et al. [43] reported that SE did not affect pregnancy loss in seasonally calving grazing dairy cows. In Norwegian Red cows, a breed with high fertility, SE at the time of first insemination was not related to late embryo loss [10]. Variable effects of SE on fertility in various studies may be due to differences in the threshold of PMNs, sample time points postpartum, and other factors affecting fertility such as breeding management, nutrition, season, breed of cows, and other diseases.

## Conclusions

In conclusion, the study showed that SE in cows clinically cured of CE reduces their fertility. The AI/P and pregnancy loss rates were statistically significantly higher in cows with SE than in cows without SE. There was a tendency towards longer interval calving to conception, lower pregnancy rate, and higher culling rate in cows with SE than in cows without SE. Further studies are necessary to confirm the impact of SE after the clinical cure of CE on fertility in dairy cows.

## Data Availability

All data generated or analysed during this study are included in this published article.

## Abbreviations

AI: Artificial insemination
CE: Clinical endometritis
PMNs: Polymorphonuclear neutrophils
PVD: Purulent vaginal discharge
SE: Subclinical endometritis
